# A20 inhibits the motility of HCC cells induced by TNF-α

**DOI:** 10.18632/oncotarget.7521

**Published:** 2016-02-20

**Authors:** Xianteng Wang, Chao Ma, Zhaoyun Zong, Ying Xiao, Na Li, Chun Guo, Lining Zhang, Yongyu Shi

**Affiliations:** ^1^ Department of Immunology, Shandong University School of Medicine, Jinan, China; ^2^ Department of Pathology, Qilu Hospital of Shandong University, Jinan, China; ^3^ Laboratory of Cellular and Molecular Medicine, Shandong University School of Medicine, Jinan, China

**Keywords:** hepatocellular carcinoma, A20, TNFAIP3, metastasis, TNF-α

## Abstract

Metastasis of hepatocellular carcinoma (HCC) can be facilitated by TNF-α, a prototypical inflammatory cytokine in the HCC microenvironment. A20 is a negative regulator of NF-κB signaling pathway. In the present study we ask whether A20 plays a role in HCC metastasis. We found that A20 expression was downregulated in the invasive cells of microvascular invasions (MVI) compared with the noninvasive cells in 89 tissue samples from patients with HCC by immunochemistry methods. Overexpression of A20 in HCC cell lines inhibited their motility induced by TNF-α. Furthermore, the overexpression of A20 inhibited epithelial-mesenchymal transition (EMT), FAK activation and RAC1 activity. By contrast, knockdown of A20 in one HCC cell line results in the converse. In addition, the overexpression of A20 restrained the formation of MVI in HCC xenograft in nude mice treated with TNF-α. All the results suggested that A20 functioned as a negative regulator in motility of HCC cells induced by TNF-α.

## INTRODUCTION

HCC is one of the most common malignancies globally, ranking fifth in incidence and third in cancer-related deaths. Despite the recent advance in diagnosis and treatment of HCC, it remains a highly lethal disease due to metastasis and consequent recurrence after curative therapies [1]. Understanding the metastatic mechanisms is a prerequisite for developing targeted molecular therapy to improve patients' survival.

HCC metastasis can be promoted by TNF-α, a critical component of the inflammatory microenvironment of HCC. TNF-α is a key mediator of inflammation and cancer. It can facilitate the metastasis of cancer by directly regulating motility of neoplastic cells [2–5]. Substantial data from clinical studies suggest that TNF-α is involved in HCC development as the serum level of TNF-α is significantly high in HCC [6]. TNF-α is also essential for spontaneous HCC development in Mdr2 −/− knockout mice [7]. Besides, TNF-αpromotes cancer metastasis after surgical operation in an experimental mouse model of liver cancer metastasis [8]. Moreover, mounting experiments *in vitro* demonstrate that TNF-α enhances cell migration via its direct effect on HCC cells [9, 10]. All the previous studies indicate that TNF-α is a prototypical inflammatory cytokine promoting HCC metastasis. However, the mechanism that can inhibit the motility induced by TNF-α is not well understood.

A20, also referred to as tumor necrosis factor alpha-induced protein (TNFAIP) 3, is an ubiquitin-editing enzyme with negative immunoregulatory function [11]. Constitutive expression of A20 is restricted in lymphoid tissues, like thymus and spleen. In A20 knockout mice, its deficiency leads to death shortly after birth due to severe inflammation and tissue damage in multiple organs. In immune cells, overexpression of A20 can terminate NF-κB signaling transduced from TNF receptors, toll-like receptors, nucleotide-binding oligomerization domain containing 2 (NOD2) receptors or T cell receptors [11, 12]. Accumulating studies find the aberrant expression of A20 in a variety of cancers. A20 is identified as a tumour suppressor in various lymphomas, as A20 gene is inactivated in these hematopoietic cancers by deletion, promoter methylation and gene mutations [12, 13]. Besides, the expression of A20 is also reduced in some epithelial cancer such as pancreatic caner [14] and colorectal tumors [15]. Moreover, A20 expression is downregulated in breast cancer brain metastases (BCBM) as compared to primary breast tumors [16]. But the relationship between A20 and HCC is rarely reported.

Based on the previous studies about the biological functions of A20 and its relevance to cancers, we asked whether A20 played an important role in the metastasis of HCC in the present study. We evaluated the A20 expression in 89 HCC specimens and found that A20 expression was down-regulated in the HCC cells invaded microvessels compared with the primary HCC cells. Gain or loss of function experiments demonstrated that A20 inhibited the motility of HCC cells induced by TNF-α. The mechanisms for the regulation of A20 in the motility of HCC cells involved EMT, FAK activation and RAC1 activity. Consistently, the overexpression of A20 in HCC cells suppressed the formation of MVI in HCC xenografts. Our findings suggested that A20 served as a inhibitor of metastasis of HCC cells induced by TNF-α.

## RESULTS

### A20 expression was decreased in the invasive HCC cells of MVI compared to noninvasive HCC cells in HCC tissue specimens

**Figure 1:**
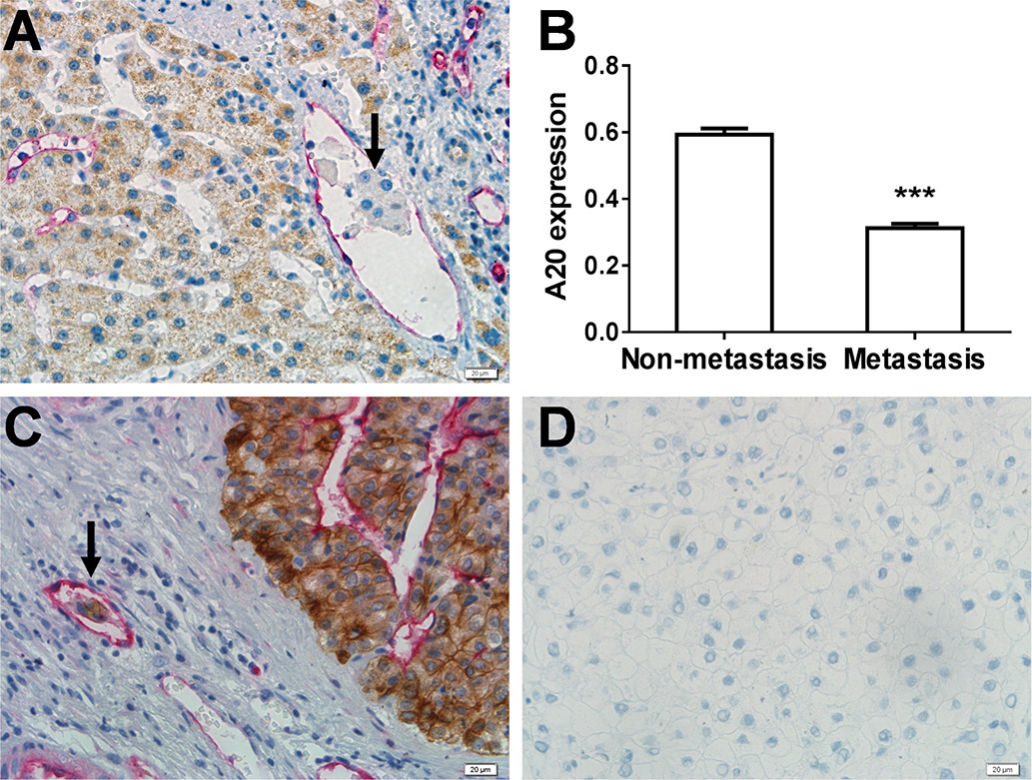
Association of downregulated expression of A20 with MVI in HCC (**A**) Examination of A20 and CD34 expression by immunohistochemistry double staining CD34-positive microvessles were stained red while A20 expression was indicated by brown color. The metastasic cells of MVI are indicated by black arrow. (**B**) Statistical analysis of the relationship between A20 expression and MVI. The A20 expression in the metastatic cells of MVI significantly decreased compared with the non-metastatic HCC cells in the 89 cases of HCC with MVI by densitometry analysis. Data shown are expressed as means ± SE. ***p < 0.001. (**C**) Identification of HCC cells in the microvessles. CK8/18 expression (brown) and CD34 expression (red) were examined by double staining immunodistochemistry to confirm that the HCC cells of MVI were HCC cells rather than other mesenchymal cells. (**D**) Negative control.

To clarify the relationship between A20 expression and HCC metastasis, we detected the expression of A20 in 89 cases of HCC specimens containing MVI by immunohistochemistry double staining technique. The A20 expression was shown in the HCC cells and developed into a brown color. The expression of CD34 was shown in endothelial cells and developed into a red color (Figure 1A). The strength of A20 expression was recorded as a value of optical density (average IOD/area). The average optical density of A20 expression in the invasive HCC cells of MVI was significantly reduced compared to that in the noninvasive cells (*p* < 0.0001, paired *T* test) (Figure 1B). CK8/18, a marker of HCC cells [17], was expressed in the invasive HCC cells as well as the primary HCC cells outside the microvessles. This confirmed that the cells invaded into mirovessels were cancer cells instead of immune cells (Figure 1C). We also examined the A20 expression in 74 cases of paired HCC tissues and adjacent non-tumor tissues by immunohistochemistry single staining technique. The average optical density of A20 expression in the HCC tissues was lower than that in the adjacent non-tumor tissues (Supplementary Figure S1).

### A20 inhibited migration of HCC cells induced by TNF-α

**Figure 2:**
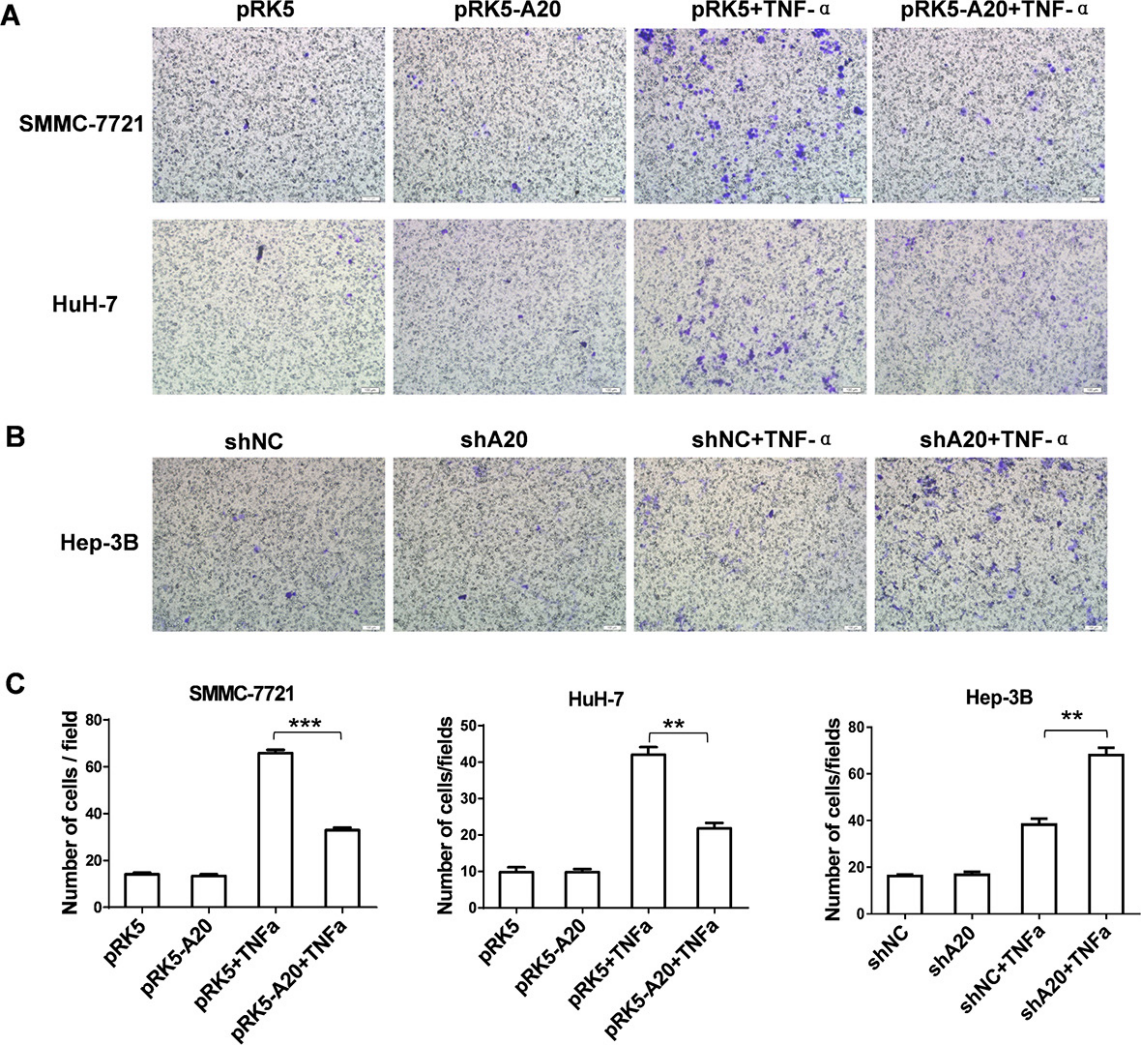
A20 inhibited migration of HCC cells induced by TNF-α (**A**) Overexpression of A20 inhibited migration of HCC cells induced by TNF-α. SMMC-7721 or HuH-7 cells were transfected with pRK5 plasmid or pRK5-A20 plasmid in the presence or absence of TNF-α. After 24 h of culture, the cells were subjected to Transwell assay. (**B**) Knockdown of A20 enhanced migration of HCC cells induced by TNF-α. Hep-3B cells were infected with shNC or shA20 in the presence or absence of TNF-α. After 24 h of culture, the cells were subjected to Transwell assay. (**C**) The number of migratory cells in the group pRK5-A20 was significantly decreased compared to the group pRK5 in the presence of TNF-α. The number of migratory cells in the group shA20 was significantly increased compared to the group shNC in the presence of TNF-α. Data are presented as means ± SE. **P < 0.01, ***P < 0.001.

As downregulation of A20 expression was associated with the MVI in HCC tissues, gain or loss of function experiments were conducted to determine the relationship between A20 expression and motility of HCC cells in the presence of TNF-α. On one hand, SMMC-7721 and HuH-7 cells were chosen to overexpress A20 by transfection of pRK5-A20 plasmids since their constitutive expression of A20 was low (Supplementary Figure S2A). The cell migration assay showed that the HCC cells with A20 overexpression presented a significant decrease in the number of migrating cells in the context of TNF-α stimulation (Figure 2A and Figure 2C). On the other hand, Hep-3B cells were used for knockdown of A20 expression by transfection of shA20 since its constitutive expression of A20 was high (Supplementary Figure S2A). With stimulation of TNF-α, the HCC cells with A20 knockdown migrated into the lower surface of the transwell membrane were increased (Figure 2B and Figure 2C). In comparison, the overexpression or knockdown of A20 did not influenced migration of the HCC cells without TNF-α. Hence, these results demonstrated that low expression of A20 enhanced migration of HCC cells induced by TNF-α.

We also performed cell invasion experiment using HuH-7 cells and BEL-7402 cells. The results showed that the overexpression of A20 restrained HCC cell invasion in the presence of TNF-α (Supplementary Figure S3).

### A20 restrained the EMT of HCC cells induced by TNF-α

**Figure 3:**
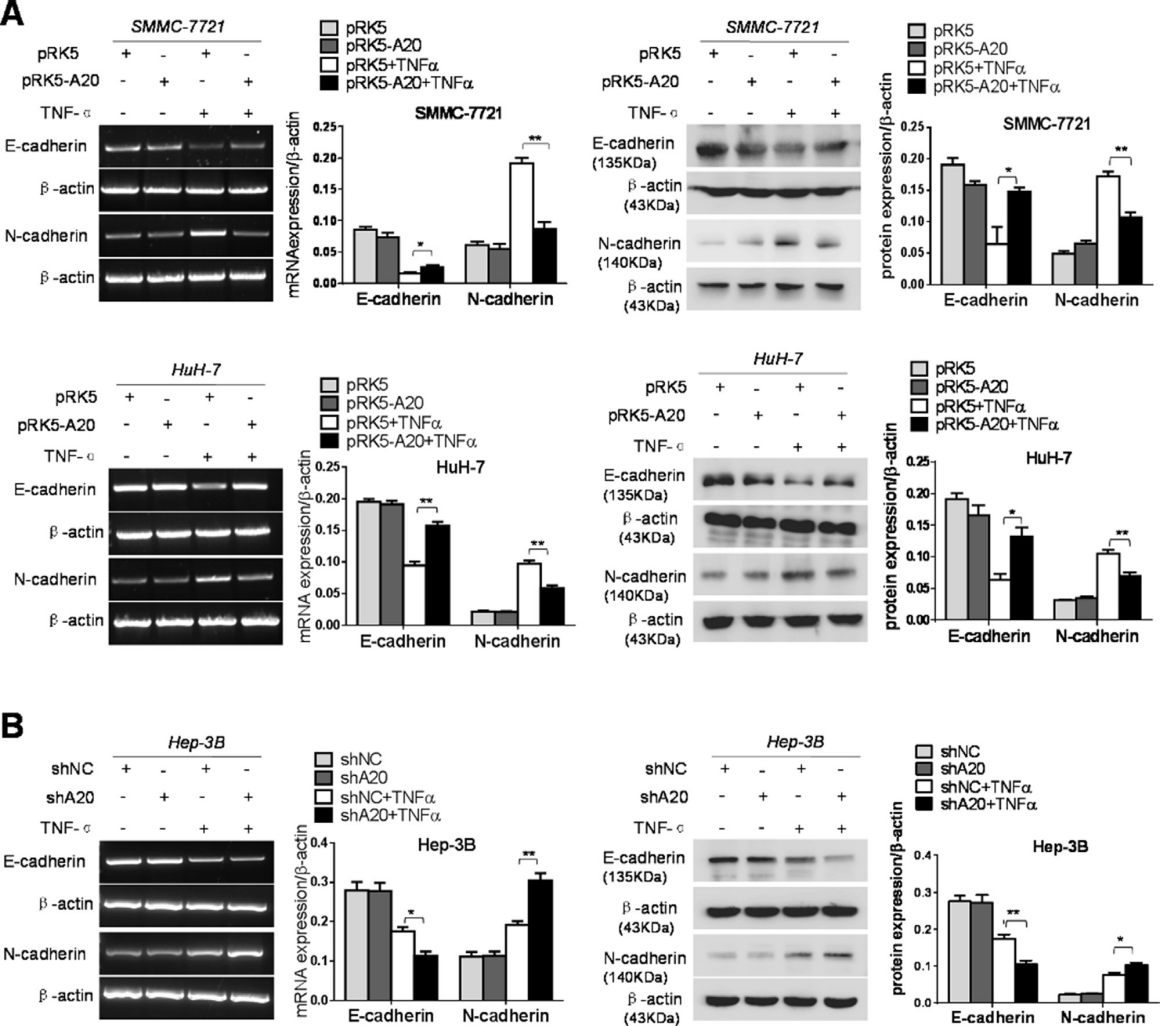
A20 restrained EMT of HCC cells induced by TNF-α (**A**) Overexpession of A20 inhibited the EMT of HCC cells induced by TNF-α. SMMC-7721 or HuH-7 cells were transfected with pRK5 plasmids or pRK5-A20 in the presence or absence of TNF-α. The increased E-acdherin expression and decreased N-cadherin expression were detected in the pRK5-A20 group compared with the pRK5 group in the presence of TNF-α. (**B**) Knockdown of A20 facilitated the EMT of HCC cells induced by TNF-α. Hep-3B cell was infected with shNC or shA20 in the presence or absence of TNF-α. The decreased E-acdherin expression and increased N-cadherin expression were detected in the shA20 group compared with the shNC group in the presence of TNF-α. Data are shown as means ± SE. *P < 0.05, **P < 0.01.

EMT is prerequisite for metastasis of epithelial cancers. The hallmarks of EMT are decreased expression of E-cadherin and increased expression of N-cadherin [3]. Therefore, we detected the expression of E-cadherin and N-cadherin in HCC cells with overexpression or knockdown of A20 in absence of TNF-α or presence of TNF-α. The expression of E-cadherin was increased and the expression of N-cadherin was decreased at both mRNA and protein level in the HCC cells transfected with pRK5-A20 compared with those transfected with pRK5 in the context of TNF-α presence (Figure 3A). In agreement, the expression of E-cadherin was decreased and the expression of N-cadherin was increased in the HCC cells transfected with shA20 compared with those transfected with shNC in presence of TNF-α (Figure 3B). By contrast, the overexpression or knockdown of A20 had little effect on the expression of E-cadherin and N-cadherin in the absence of TNF-α. These results suggested that low expression of A20 in HCC cells promoted their EMT induced by TNF-α.

**Figure 4:**
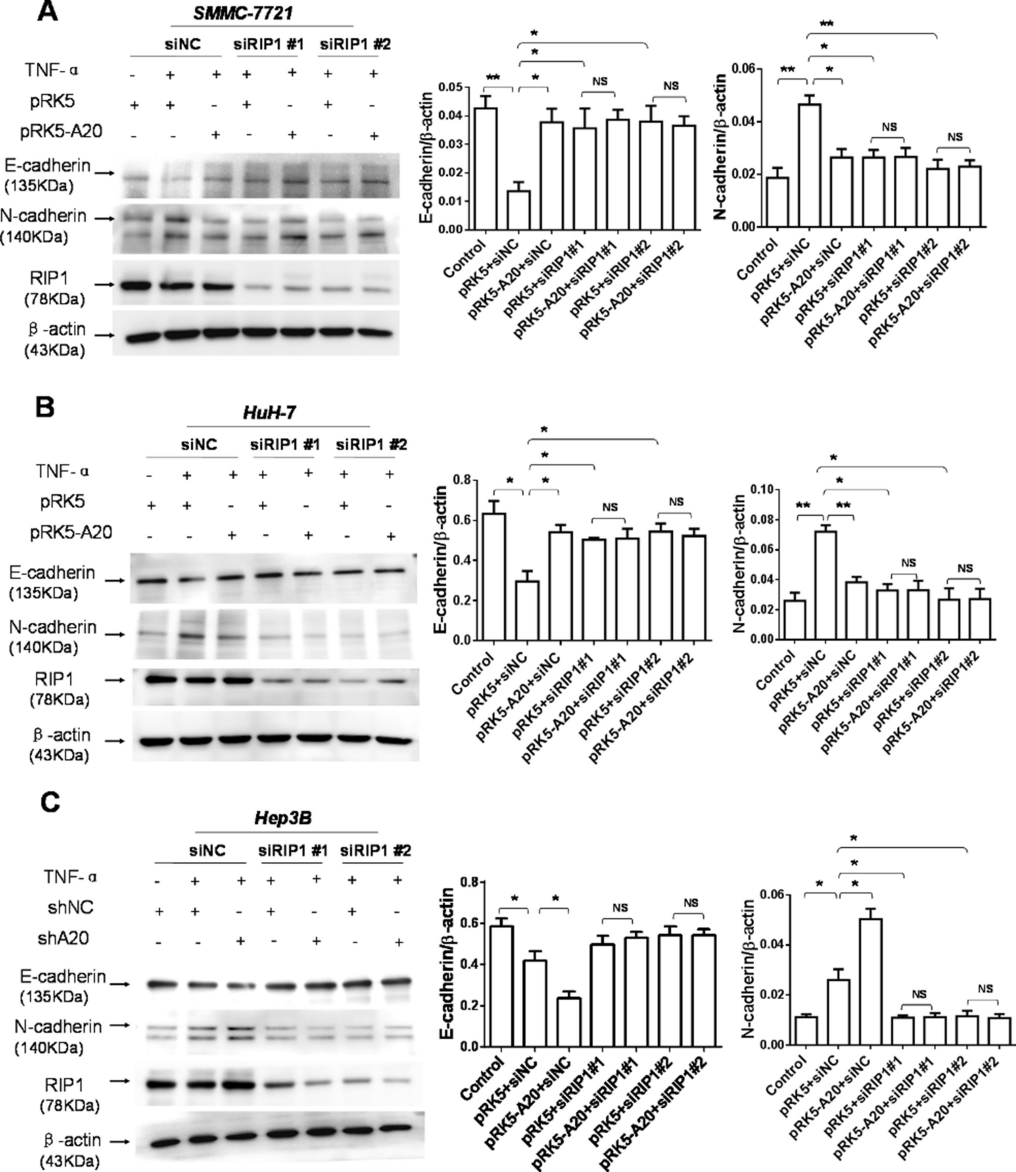
The inhibition of A20 in the EMT of HCC cells induced by TNF-α was dependent of RIP1 (**A**) Knockdown of RIP1 abrogated the inhibitory effect of A20 overexpession on the expression of E-cadherin and N-cadherin in the SMMC-7721 cells in the presence of TNF-α. (**B**) Knockdown of RIP1 abrogated the inhibitory effect of A20 overexpession on the expression of E-cadherin and N-cadherin in the HuH-7 cells in the presence of TNF-α. (**C**) Knockdown of RIP1 abrogated the promoting effect of A20 knockdown on the expression of E-cadherin and N-cadherin in the Hep-3B cells in the presence of TNF-α. Data are shown as means ± SE. *P < 0.05, **P < 0.01, ***P < 0.001.

As the deubiquitination of RIP1 by A20 contributes to inhibitory effect of A20 on the TNF signaling [18], we asked whether the inhibitory effect of A20 on the EMT induced by TNF-α was attributed to the interaction between A20 and RIP1 in HCC cells. Silenced expression of RIP1 by RIP1 siRNA abrogated the inhibitory effect of A20 on the EMT induced by TNF-α, suggesting that the RIP1 was associated with the inhibitory effect of A20 on the EMT induced by TNF-α (Figure 4). The overexpression of A20 in HCC cells attenuated the K63-linked and K-48 ubiquitination of RIP1 in the presence of TNF-α (Supplementary Figure S4). These results implied that the inhibitory effect of A20 on the EMT induced by TNF-αmay be mediated by its catalyzing deubiquitination of RIP1 in HCC cells.

### A20 inhibited the activation of FAK in HCC cells induced by TNF-α

**Figure 5:**
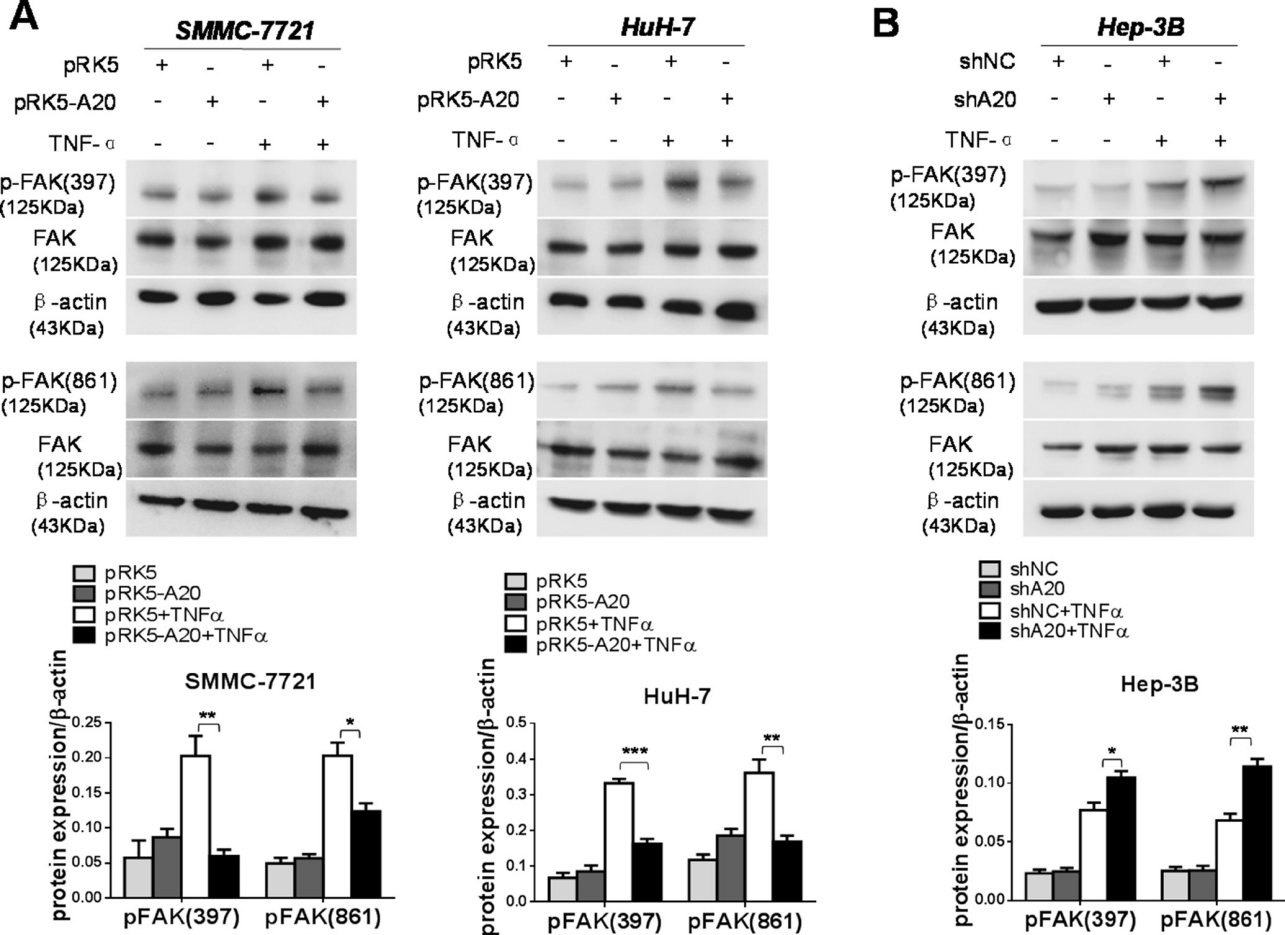
A20 suppressed the activation of FAK induced by TNF-α in HCC cells (**A**) Overexpession of A20 inhibited the FAK activation induced by TNF-α. SMMC-7721 or HuH-7 cells were transfected with pRK5 plasmids or pRK5-A20 plasmids in the presence or absence of TNF-α. The expression of p-FAK (397) and p-FAK (861) was decreased in the pRK5-A20 group compared with the pRK5 group in the presence of TNF-α. (**B**) Knockdown of A20 enhanced the FAK activation induced by TNF-α. Hep-3B cell was infected with shNC or shA20 in the presence or absence of TNF-α. The expression of p-FAK (397) and p-FAK (861) was increased in the shA20 group compared with the shNC group in the presence of TNF-α. Data shown are means ± SE. *P < 0.05, **P < 0.01, ***P < 0.001.

FAK is a non-receptor protein tyrosine kinase, the activity of which elicits the turn-over of cell contacts with the extracellular matrix, promoting cell migration [19]. Hence, we investigated the role of A20 in activation of FAK induced by TNF-α in HCC cells. We found that overexpression of A20 reduced the phosphorylation of FAK in HCC cells stimulated with TNF-α (Figure 5A). Consistently, silence of A20 expression by A20 shRNA elevated the activation of FAK in Hep-3B cells in presence of TNF-α (Figure 5B). Furthermore, silenced expression of FAK by FAK siRNA attenuated the enhancement of cell motility by knockdown of A20 expression using shA20 in Hep-3B cells treated with TNF-α (Supplementary Figure S5). This result linked the suppressive effect of A20 on the FAK activation to the inhibition of A20 on the cell motility.

**Figure 6:**
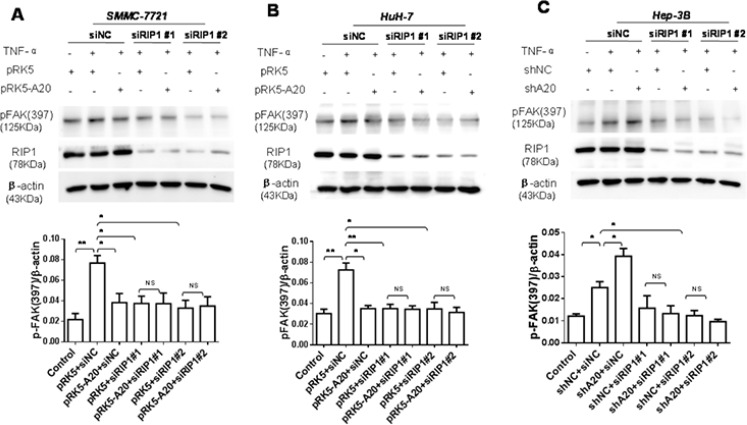
The inhibition of A20 in the FAK activation induced by TNF-α depended on RIP1 (**A**) and (**B**) SMMC-7721 or HuH-7 cells were transfected with siNC and pRK5 or pRK5-A20, or siRIP1 and pRK5 or pRK5-A20 in the presence or absence of TNF-α. The cell lysates were subjected to Western blot. Knockdown of RIP1 abrogated the inhibitory effect of A20 overexpession on the expression of p-FAK (397) in the HCC cells in the presence of TNF-α. (**C**) Hep-3B cells were transfected with siNC and shNC or shA20, or siRIP1 and shNC or shA20 in the presence or absence of TNF-α. The cell lysates were subjected to Western blot. Knockdown of RIP1 abrogated the promoting effect of A20 knockdown on the expression of p-FAK (397) in the Hep-3B cells in the presence of TNF-α. Data are shown as means ± SE. *P < 0.05, **P < 0.01.

Subsequently, we asked how A20 impact the activation of FAK. Previous studies demonstrate that TNF-α signaling can result in association of RIP1 with FAK [20]. Thus, we speculated that the inhibition of A20 on the activation of FAK might depend on RIP1 protein. To test this notion, we used RIP1 siRNA to silence the expression of RIP1. In the context of TNF-α stimulation, the knockdown of RIP1 decreased the activation of FAK induced by TNF-α in HCC cells. The RIP1 knockdown abrogated the promoting effect of A20 knockdown on the activation of FAK (Figure 6). These results suggested that RIP1 was potential target of A20 for its inhibitory effect on FAK activation induced by TNF-αin HCC cells. The overexpression of A20 in HCC cells attenuated the K63- and K48-linked ubiquitination of RIP1 in the presence of TNF-α (Supplementary Figure S4). Taken together, all the results implied that the inhibitory effect of A20 on the FAK activation induced by TNF-α may be mediated by its catalyzing deubiquitination of RIP1 in HCC cells.

### A20 suppressed the activity of Rac1 in HCC cells induced by TNF-α

**Figure 7:**
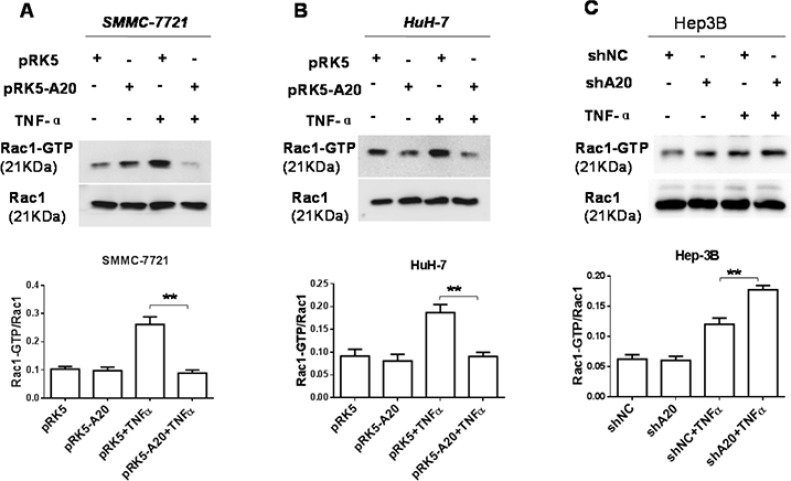
A20 suppressed the activity of Rac1 in HCC cells induced by TNF-α (**A**) and (**B**) SMMC-7721 or HuH-7 cells were transfected with pRK5 plasmids or pRK5-A20 plasmids in the presence or absence of TNF-α. The cell lysates were subjected to pull-down assay by GST-PAK1-PBD fusion protein. The overexpression of A20 in the HCC cells suppressed the activity of Rac1 induced by TNF-α. (**C**) Hep-3B cells were transfected with shNC or shA20 in the presence or absence of TNF-α. The cell lysates were subjected to pull-down assay by GST-PAK1-PBD fusion protein. The knockdown of A20 in the HCC cells enhanced the activity of Rac1 induced by TNF-α. Data are shown as means ± SE. **P < 0.01.

The small Ras-like GTPases Rac1 controls cell motility by regulating actin cytoskeleton reorganization to form cell surface extensions (lamellipodia) [21], the activity of which can be stimulated by TNF-α [22]. Hence, we asked whether A20 could regulate the activation of Rac1 induced by TNF-αin HCC cells. The results showed that the overexpression of A20 by transfection of pRK5-A20 plasmids reduced the level of Rac1-GTP in HuH-7 and SMMC-7721 cells stimulated with TNF-α (Figure 7). Consistently, silence of A20 expression by A20 shRNA elevated the level of Rac1-GTP in Hep-3B cells in presence of TNF-α. These results indicated that A20 inhibited Rac1 activity stimulated by TNF-α.

Silenced expression of Rac1 by Rac1 siRNA abrogated the enhancement of cell motility by A20 knockdown in Hep-3B cells treated with TNF-α (Supplementary Figure S5). In addition, A20 expression also inhibit the formation of ruffling lambellipodia in HCC cells (Supplementary Figure S6). Collectively, these results linked the suppressive effect of A20 on the Rac1 activity to the inhibition of A20 on the cell motility in the presence of TNF-α.

Then, we asked whether the inhibitory effect of A20 on the Rac1 activity was associated RIP1. Silenced expression of RIP1 by RIP1 siRNA suppressed the RAC1 activation and abrogated the inhibitory effect of A20 on the RAC1 acivation induced by TNF-α. This result suggested that RIP1 was associated with the inhibitory effect of A20 on Rac1 activation (Supplementary Figure S7). The overexpression of A20 in HCC cells attenuated the K63- and K48-linked ubiquitination of RIP1 in the presence of TNF-α (Supplementary Figure S4). Collectively, all the results implied that the inhibitory effect of A20 on the Rac1 activity induced by TNF-α may be mediated by its deubiquitinizing RIP1 in HCC cells.

### Overexpression of A20 in HCC xenograft restrained MVI induced by TNF-α

**Figure 8:**
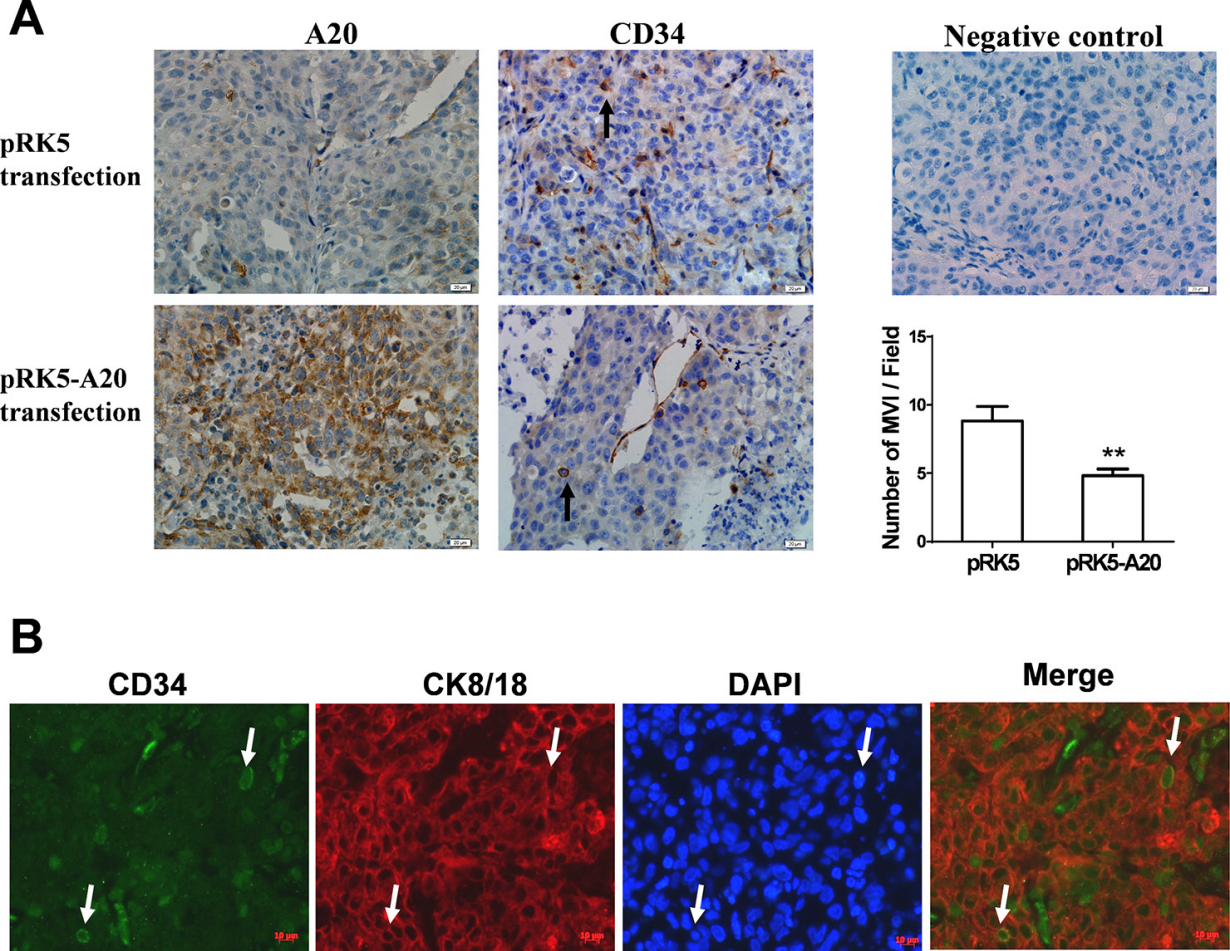
Overexpression of A20 inhibited MVI in HCC xenograft (**A**). The inhibition of A20 in formation of MVI in HCC xenograft tumor in the context of TNF-αpresence. The xenograft tumors derived from HCCLM3 cells were sectioned and stained for the expression of A20 or CD34 by immunohistochemistry. A20 was overexpressed in xenograft tumors transfected with pRK5-A20 plasmids. The number of MVI (inidicated by the black arrows) in the xenograf tumors of pRK5-A20 group (*n* = 5) was significantly less than pRK5 group (*n* = 5). Data shown are expressed as means ± SE. **p < 0.01. (**B**). Identification of the HCC cells invading microvessels. CD34 expression (green) and CK8/18 expression (red) were examined by double staining immunofluorescence. The neuclei were stained blue with DAPI. The merge image showed that CK8/18 positve cells located in the CD34-positve microvessles (indicated by white arrows).

To further verify the relevance of A20 expression to metastasis of HCC cells, we established subcutaneous xenograft tumor model using HCCLM3 cells in nude mice. When visible tumor appeared, the mice were injected with empty pRK5 plasmids or recombinant pRK5-A20 plasmids, as well as TNF-α. The sections from the xengraft tumors were stained with anti-CD34 antibody. The MVI consisted of microvessels and invasive cells (The invasive cells are inside the microvessels). The average number of MVI in the pRK5-A20 group was decreased significantly compared with the pRK5 group (Figure 8A). In addition, the cells invading microvessels in the xenograft tumor was CK8/18 positive. This confirmed that the cells of MVI were HCC cells rather than other mesenchymal cells (Figure 8B). All the results suggested that overexpression of A20 suppressed the metastasis of HCC cells in the context of TNF-α presence.

## DISCUSSION

The molecular mechanisms of HCC metastasis are elusive. In the present study, we investigated the relationship between A20 expression and metastasis of HCC. We found that the expression of A20 was reduced in malignant cells invading into microvessles compared with the non-metastatic cells outside the microvessles in human HCC tissues. A20 suppressed motility of HCC cells by inhibition of EMT, FAK activation and Rac1 activity induced by TNF-α *in vitro*. Overexpression of A20 in HCC xenograft decreased the occurrence of MVI in the presence of TNF-α. All the results suggested that A20 may negatively regulate the motility of HCC cells in the inflammatory microenvironment.

Alteration of A20 expression implies its role in cancer pathogenesis. The expression of A20 is constitutive in lymphoid tissues and inducible in other cells such as endothelial cells, islet cells and bronchial epithelial cells, as well as a variety of tumor cells [23]. The expression profile of A20 in cancers is cell type dependent. High expression of A20 was found in some solid cancers such as bladder cancer [24], nasopharyngeal carcinoma [25], and squamous cell carcinoma [26]. On the other hand, downregualtion of A20 expression was discovered in lymphomas and some solid cancer such as pancreatic caner and colorectal tumor. Furthermore, the downregulation of A20 may be involved in tumor invasion and metastasis since it is associated with breast cancer brain metastases [16] and invasion of salivary adenoid cystic carcinoma cells [27]. Previous studies show that A20 expression is absent in normal liver, but is present in hepatitis tissue [28, 29]. A20 is also expressed in HCC tissues probably due to the inflammatory environment of HCC [30]. The data deposited in the GEO profile database shows that the A20 mRNA level in HCC with metastasis is decreased compared to that in HCC without metastasis (http://www.ncbi.nlm.nih.gov/geoprofiles/48364228). Consistent with these previous studies, our results showed that A20 was expressed in HCC. The A20 expression was elevated in the adjacent non-tumor tissues compared to the HCC tissues. Furthermore, we found that A20 expression was decreased in the HCC cells of MVI compared with the primary HCC cells. This finding implicated A20 in HCC metastasis since MVI formation is related to the metastasis and recurrence of HCC [31, 32].

RIP1 may be a key factor for the mechanism, by which A20 inhibited the motility of HCC cell induced by TNF-α. First, we found that A20 inhibited the EMT, FAK activation and RAC1 activity in HCC cells treated with TNF-α. Second, knockdown of RIP1 blocked the EMT, FAK activation and RAC1 activity induced by TNF-α. That is, RIP1 was required for the molecular events associated with cell motility in the presence of TNF-α. Third, A20 can not only ubiquitinate but also deubiquinate RIP1 to regulate RIP1 function [18, 33, 34]. Our results showed that the overexpression of A20 deubiquitinated RIP1 in the context of TNF-α presence. Thus, all these results implied that the deubiquitination of RIP1 may mediated the inhibitory effect of A20 on the EMT, FAK activation and RAC1 activity induced by TNF-α. However, more supports are needed to establish the conclusion. Especially, direct proof for the relation of deubiquitination of RIP1 to the EMT, FAK activation and RAC1 activity induced by TNF-αneeds to be provided in the future research.

Our research showed that the downregulation of A20 in the metastatic cells of MVI in HCC specimens and demonstrated that downregulation of A20 in HCC cells enhanced the cell motility induced by TNF-α. These findings support a notion that collaboration of genetic or epigenetic alteration of the cancer cells with heterotypic signals from the tumor environment is required for the cancer progression. The genetic and epigenetic alterations acquired by carcinoma cells during primary tumor formation are likely to increase their responsiveness to various contextual signals such as TGF-β, TNF-α, IL-6 and EGF [35]. For instances, cancer cells that have lost p53 function are more responsive to EMT-inducing signals [36]. Besides, TGF-β and Ha-Ras cooperatively increase metastasis of epithelial tumor cells [37]. As for the present study, the doweregulation of A20 in HCC cells of MVI may collaborate with the heterotypic signal of TNF-α to promote invasion and metastasis of HCC cells.

Our results should be interpreted with caution. There are some limitations in the present study. Our results showed that A20 expression was downregulated in the invasive cells of MVI in HCC tissues. Knockdown of A20 significantly enhanced the motility of HCC cells in the context of TNF-α presence. But the TNF-α expression in the HCC tissues was not validated. It was assumed that TNF-α was expressed in the HCC tissues based on previous studies [38, 39]. We will examine the TNF-α expression as well as the relationship between TNF-α expression and MVI in our future research. Although our results showed that A20 inhibited the EMT, FAK activation and RAC1 activity, the inhibitory effect of A20 was modest. More effort will be put in our future research to explore whether there may be additional mechanisms underlying the inhibitory effect of A20 on the HCC cell motility.

In summary, we found the relevance of A20 expression to metastasis of HCC. The A20 expression was decreased in the malignant cells of MVI. Further experiments *in vitro* demonstrated that the overexpression of A20 restrained motility of the HCC cells in the presence of TNF-α. The molecular mechanisms involved in the inhibition of A20 in EMT, activation of FAK and Rac1 activity. Experiment of xenograft tumor *in vivo* verified that the overexpression of A20 suppressed microvascular metastasis of HCC cells. Our findings suggested that A20 functioned as a negative regulator in the motility of HCC cells induced by TNF-α.

## MATERIALS AND METHODS

### Clinical specimens and immunohistochemistry double staining assay

Eighty-nine cases of human HCC specimens with MVI were selected by a pathologist from 106 cases of HCC specimens (archived paraffin-embedded sections) from Qilu hospital, Shandong University. All the patients gave their written informed consent. The medical ethical committee of Shandong University approved this study.

To observe the expression of A20 or CK8/18 in the HCC cells invaded into microvessels in the HCC specimens, double staining immunohistochemisty was performed on a 4-mm thick section using a two-step method with a primary antibody mixture and a seconday antibody mixture. The primary antibody mixture was consisted of a mouse anti-CD34 antibody (Catalog number ZM-0046, 1:70 dilution; ZSGB-BIO, China) paired with a rabbit anti-A20 antibody (Catalog number 2644–1, 1:500 dilution; Epitomics, USA), or a mouse anti-CK8/18 antibody (Catalog number ZM-0315, 1:200 dilution, ZSGB-BIO, China) paired with a rabbit anti-CD34 antibody (Catalog number ab81289, 1:300 dilution, Abcam, China). The secondary antibody mixture includes a goat anti-rabbit IgG antibody conjugated to horseradish peroxidase (HRP) paired with a goat anti-mouse IgG antibody conjugated to alkaline phosphatase (AP), or a goat anti-mouse IgG antibody conjugated to HRP paired with a goat anti-rabbit IgG antibody conjugated to AP (Kit catalog number DS-0001 or DS-0005, ZSGB-BIO, Beijing, China). The staining operation was performed as described previously [40]. Negative controls were obtained by omiting the primary antibodies.

The A20 expression in HCC cells was evaluated by a digital image system. In each section, one to three images of representative fields with MVI were captured at a magnification of ×400 and saved as TIFF files. The images were analyzed with software Image-Pro Plus version 6.0. The invasive HCC cells of MVI (more than 3 cells) or the noninvasive HCC cells outside the microvessels (more than 100 cells) were selected using selection tool in the software. The area and the integrated optical density (IOD) of the positive staining of A20 were measured and the mean density is calculated as IOD/area for the invasive or noninvasive HCC cells respectively. The mean density (one image) or average of mean densities (more than one image) for the invasive HCC cells or the noninvasive HCC cells in each slide was used to represent an individual sample.

### Cells and treatments

HCC cell lines (SMMC-7721, HuH-7 and Hep-3B) were purchased from the Cell Bank of Type Culture Collection of Chinese Academy of Science (CBTCCCAS), Shanghai, China. HCC cell line HCCLM3 was from China Center for Type Culture Collection, Wuhan, China. All the cells were maintained in Dulbecco's modified Eagle's medium (DMEM) supplemented with 10% heat-inactivated fetal bovine serum at 37°C with 5% CO2.

Human A20 gene was amplified from human peripheral blood mononuclear cells by RT-PCR and cloned into plasmids pRK5. Specific A20 shRNA (shA20) (sense 5′ CACCGAAGTGGACTTCAGTACAATTCAAGAGATT GTACTGAAGTCCACTTCTTTTTTG 3′ and antisense 5′ GATCCAAAAAAGAAGTGGACTTCAGTACAAT CTCTTGAATTGTACTGAAGTCCACTTC 3′), negative control shRNA (shNC) (sense 5′ CACCGTTCTCCGAA CGTGTCACGTCAAGAGATTACGTGACACGTTCGG AGAATTTTTTG 3′ and antisense 5′GATCCAAAAAATT CTCCGAACGTGTCACGTAATCTCTTGACGTGACAC GTTCGGAGAAC 3′), RIP1 siRNA (siRIP1) #1 (sense 5′ GCCAGCUGCUAAGUACCAATT 3′ and antisense 5′ UUGGUACUUAGCAGCUGGCTT 3′) siRIP1 #2 (sense 5′ GCAAAGACCUUACGAGAAUTT 3′ and antisense 5′ AUUCUCGUAAGGUCUUUGCTT 3′) and negative control siRNA (siNC) (sense 5′ UUCUCCGAACGUGUC ACGUTT 3′ and antisense 5′ ACGUGACACGUUCGGAG AATT 3′) were purchased from GenePharma, China. Transfection of HCC cells with plasmids or siRNA was operated using Lipofectamine2000 according to the manufacturer's protocols (Life Technologies, USA). After the transfection, the HCC cells were treated with TNF-α at a concentration of 50 ng/ml for 24 h. The total RNA or protein was extracted for RT-PCR or Western blot assay.

### Transwell cell migration assay

After transfection, HCC cells were cultured for 24 h with or without TNF-α (50 ng/ml). These cells were suspended in serum-free medium with TNF-α (50ng/ml) and seeded into the upper chamber of a Transwell apparatus with an 8 um pore size membrane (Millipore, Bedford, USA). The lower compartments were filled with medium containing 10% FBS and TNF-α (50 ng/ml). After 24 h of incubation, the cells migrated to the bottom surface of the membrane were stained with 0.5% crystal violet, examined by microscopy. Values for migration were expressed as the average number of migrated cells per microscopic field (×100) over five fields. All the assays were repeated at least twice.

### RT-PCR analysis

Total RNA was extracted from the HCC cells and reversely transcribed into cDNAs using Reverse Transcription System (Tiangen Biotech, China). Equal amounts of cDNA for each sample were used as template for PCR. The sequence of the forward and the reverse primers were as follows. β-actin gene: 5′ AGGCCAA CCGCGAGAAGATG 3′ and 5′ CACACGGAGTACTTG CGCTCAG 3′; E-cadherin gene: 5′ CTCGGCCTGAAGTGA CTCGTAAC 3′ and 5′ CAGCAACGTGATTTCTGCATTTC 3′; N-cadherin gene: 5′ TGTGGAGCCTGATGCCATCAAG 3′ and 5′ AGCCTATGCCAAAGCCTCCAGC 3′; RIP1 gene: 5′ GTGGTACCATTTTGGGCGTTC 3′ and 5′ CGGAG TACTCATCTCGGCTTT 3′. The density of the band from electrophoresis of the PCR products was quantified by densitometry using GelPro 3.2 software. The expression level of each gene was represented as density ratios of their mRNA amount relative to β-actin mRNA content in each individual sample.

### Western blot assay

The cell lysates from each sample was subjected to electrophoresis and transferred onto a polyvinylidene difluoride membrane. The membrane was probed with primary antibodies and HRP-conjugated secondary antibodies, and then developed using enhanced chemiluminescence (ECL) system (Beyotime Biotechnology, China). The density of the bands was quantified by densitometry using GelPro 3.2 software. The β-actin protein served as internal control to normalize the expression level of other proteins.

The primary antibodies included an anti-RAC1 antibody (Catalog number 8631, 1:1000 dilution, Cell Signaling Technology, USA), an anti-A20 antibody, an anti-FAK Phospho (pY397) antibody, an anti-FAK Phospho (py861) antibody, an anti-FAK antibody, an anti-E-Cadherin antibody and an anti-N-Cadherin antibody (Catalog number 2644–1, 2211-S, 2153–1, 1700-S, 1702–1 and 2447–1, 1:1000 dilution, Epitomics, USA), an anti-RIP1 antibody (Catalog number ab125072, 1:1000 dilution, Abcam, China), and an anti-β-actin antibody (Catalog number PR-0255, 1:2000 dilution, ZSGB-BIO, China). The secondary antibodies included a goat anti-rabbit IgG antibody and a goat anti-mouse IgG antibody (Catalog number ZB-2301 and ZB-2305, 1:2000 dilution, ZSGB-BIO, China).

### Rac1 activation assay

After transfection and TNF-α treatment of HCC cells, Rac1 pull-down assays were performed according to manufacturer's instruction (Catalog number 8815, Cell Signaling Technology, USA). GST-PAK1-PBD fusion protein beads were used to enrich active GTP-bound Rac1. The proteins on the beads or the total cell lysates were subjected to western blot. The expression level of active Rac1-GTP was quantified by GelPro 3.2 software relative to the total Rac1 content.

### Xenograft tumor assay

Experiments involving animals were approved by the Institutional Animal Care and Use Committee of Shandong University. Five-week-old male BALB/c nude mice (Huafukang Biotechnology Ltd, Beijing, China) were subcutaneously injected with HCCLM3 cells (1 × 10^7^) in the left flank region. When visible tumor appeared, the mice were randomly divided into pRK5 plasmid group (*n* = 5) and pRK5-A20 plasmid group (*n* = 5). Then, each mouse was injected with 10 μg of pRK5 plamid or pRK5-A20 plamid in 50 μl transfection reagent (Polyplus-transfection Inc, New York, USA), as well as 1 μg of TNF-α in the tumor every two days. The mice were sacrificed ten days later and the tumors were isolated to prepare paraffin sections for immunohistochemistry single staining assay and immunofluorescence double staining assay.

### Immunohistochemistry single staining assay

To evaluate the expression of A20 or MVI in the HCC xenograft from nude mice, the paraffin slides made from the xengraft tumor were stained with a rabbit anti-human A20 antibody (Catalog number 2644–1, 1:200 dilution, Epitomics, USA) or a rabbit anti-mouse CD34 anitibody (Catalog number ab81289, 1:300 dilution, Abcam, China) at 4°C overnight. Secondary staining was performed with HRP-conjugated anti-rabbit IgG using a MaxVsion Kit and 3, 5-diaminobenzidine (DAB) peroxidase Substrate Kit (Maixin Co., China). The sections were counterstained with hematoxylin. Negative controls were performed by omiting the primary antibodies. The number of MVI was counted manually by microscopy at high-power field (×400). The number of MVI in each sample was determined by averaging the number of MVI in 3 high-power fields in the slide.

### Immunofluorescence double staining assay

The sections from xenograft tumors were stained with a primary antibody mixture containing a mouse anti-human CK8/18 (Catalog number ZM-0315, 1:200 dilution, ZSGB-BIO, China) and a rabbit anti-mouse CD34 (Catalog number ab81289, 1:300 dilution, Abcam, China) at 4°C overnight. Then, a secondary antibody mixture containing a goat anti-rabbit IgG labeled with Alexa Fluor 488 and a goat anti-mouse IgG labeled with TRITC was incubated at room temperature for 30 minutes. Finally, Nuclei were stained by DAPI (Beyotime Biotechnology, China) for 5 min. The slides was mounted with antiquench mounting medium and subjected to observation by fluorenscence microscopy.

### Statistical analysis

The statistical analysis was performed with the GraphPad Prism software, version 5.0. The statistical significance of differences between two experiment groups was determined by Student's unpaired or paired *t*-test. Values of *P* < 0.05 were considered statistically significant.

## SUPPLEMENTARY MATERIALS FIGURES


