# Supplementary material for: A20 inhibits the motility of HCC cells induced by TNF-α
Source: Oncotarget. 2016 Feb 20;7(12):14742–54. doi: 10.18632/oncotarget.7521 (PMC4924748; doi:10.18632/oncotarget.7521)
Supplement: Supplementary file 1 [file oncotarget-07-14742-s001.pdf]

A20 inhibits the motility of HCC cells induced by TNF-α

Supplementary Materials

MATERIALS AND METHODS

Immunohistochemistry

Immunohistochemistry assay was used to measure the expression of A20 in 74 cases of HCC specimens paired with the adjacent non-tumor tissues (archived paraffin-embedded sections) from Shanghai Outdo Biotech Company, China. All the patients gave their written informed consent. The medical ethical committee of Shandong University approved this study.

Deubiquitination assays

For analysis of the deubiquitinating effect of A20 on the ubiquitination of RIP1, HuH-7 cells were cotransfected with plasmid pRK5-A20 (or pRK5 vector control) and pCMV-HA-Ub wild type (WT) or pCMV-HA-Ub mutants (K48 or K63). The cells were lysed in immunoprecipitation buffer containing 1.0% (v/v) Nonidet P-40, 50 mm Tris-HCl, pH 7.4, 50 mm EDTA, 150 mm NaCl, and a protease inhibitor mixture. After centrifugation for 10 min at 14,000 × g, the supernatants were incubated with anti-RIP1 for 1 hour, and then, incubated with protein A/G Plus-agarose immunoprecipitation reagent (Santa Cruz Biotechnology, USA). After 18 hours of incubation, agarose beads were washed five times with immunoprecipitation buffer. Immunoprecipitates were eluted by boiling with 1% (w/v) SDS sample buffer. Immunoprecipitates or total cell lysates were analyzed by Western blot analysis with ECL detection reagents.

Transwell cell invasion assay

HCC cells were transfected with pRK5-A20 or pRK5 plasmids and cultured with 50ng/ml of TNF-α for 24 hours. The cells in serum-free medium were put in the upper chamber of the transwell apparatus precoated with Matrigel (BD Biosciences, USA) to form matrix barriers. The lower chamber was filled with 10% FBS medium. After 24 hours of incubation, the cells migrated into the bottom surface of the transwell membrane were stained with cystal violet and counted by microscopy.

Fluorescence detection of actin filament

HCC cells (BEL7402) were seeded on cover slips in a 24-well plate and transfected with plasmid PRK5 or PRK5-A20 using Jet-Prime transfection reagent (Polyplus transfection, France). A scratch was made across the confluent monolayer of cell culture using a 10μl pipette tip. After 24 hours of culture, the cells on the cover slip were fixed, permeabilized and then stained with Tetramethylrhodamine(TRITC)-conjugated phalloidin (Sigma–Aldrich, USA) for 1h. Nuclei were stained by 4',6-diamidino-2-phenylindole (DAPI) (Beyotime, China) for 5 min. The lambelipodia in the cells located at the edge of the scratch was counted by fluorescence microscopy. The number of lambelipodia per cell for the PRK5-A20 group was significantly less than that for the PRK5 vector control group.

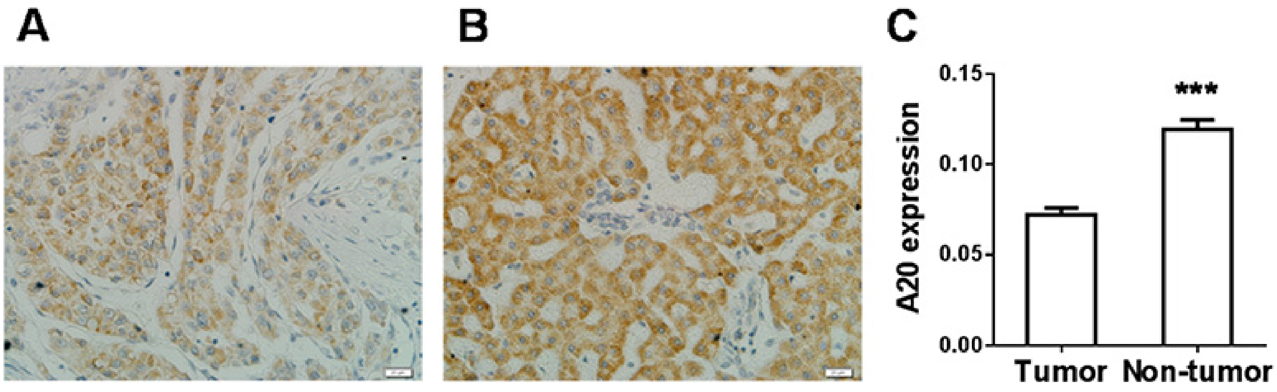

Supplementary Figure S1: Downregulation of A20 expression in HCC tissues compared to the adjacent non-tumor tissues. (**A**) The expression of A20 in HCC tissues. (**B**) The expression of A20 in non-tumor liver tissues. (**C**) The expression of A20 in HCC was downregulated compared to the non-tumor tissues by densitometry analysis. Data shown are expressed as means ± SE. ***$p < 0.001$.

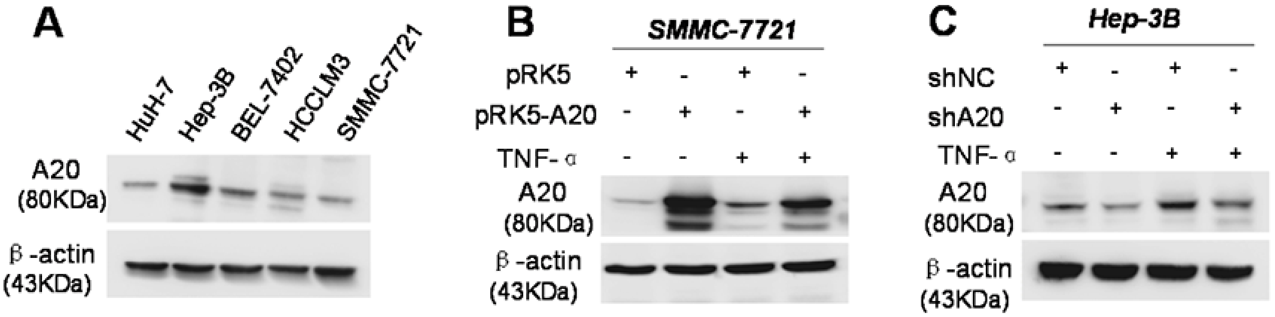

Supplementary Figure S2: Endogenous and exogenous expression of A20 in HCC cell lines. (**A**) Endogenous expression of A20 in various HCC cell lines. (**B**) Overexpression of A20 in SMMC-7721 cells transfected with plasmid pRK5-A20. (**C**) Knockdown of A20 expression in Hep-3B cells with shA20.

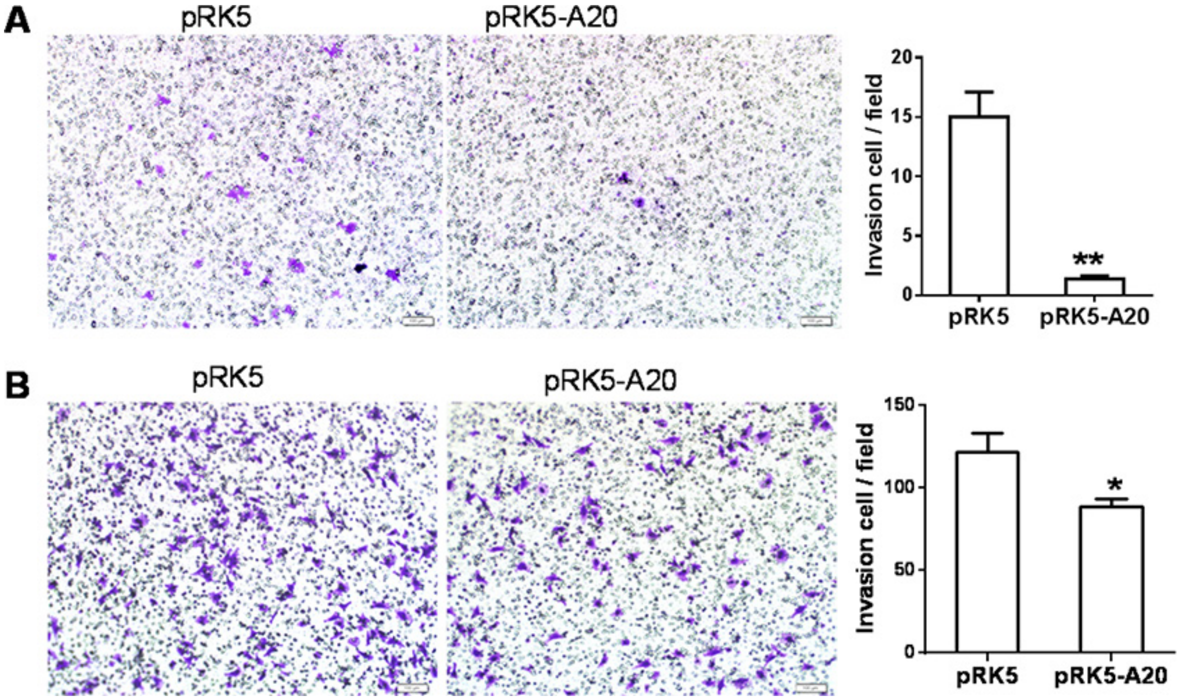

Supplementary Figure S3: A20 inhibits the invasion of HCC cells in the presence of TNF-α in the presence of TNF-α. HuH-7 cells (**A**) or BEL7402 cells (**B**) were transfected with PRK5-A20 or PRK5 plasmids in the presence of TNF-α. The cells were subjected to Transwell invasion assay. The cells that migrated to the bottom surface of the Transwell membrane precoated with Matrigel in the PRK5-A20 group are significantly less than that of PRK5 group. $*p < 0.05$. $**p < 0.01$.

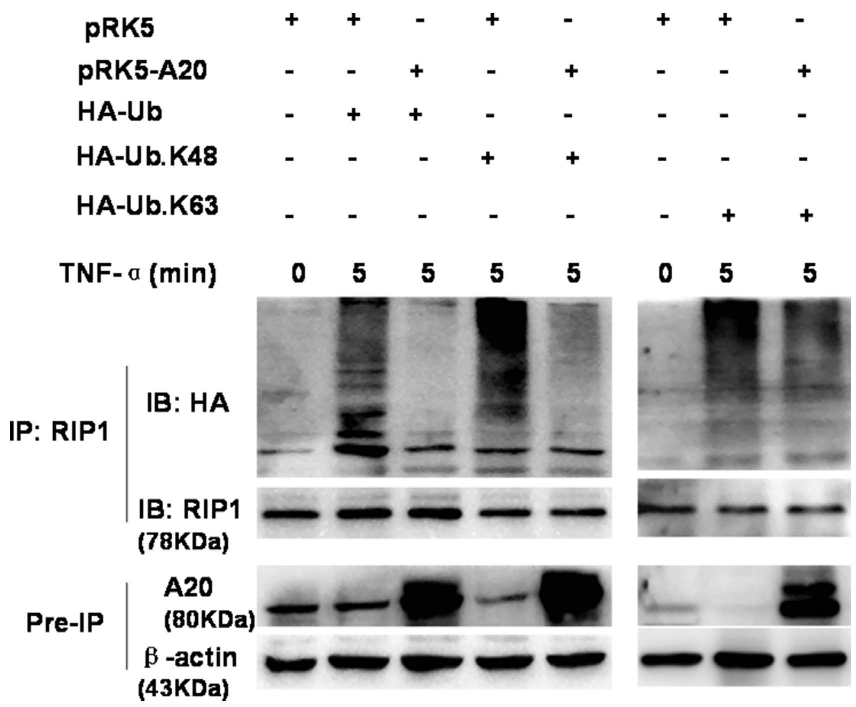

Supplementary Figure S4: Deubiquitination of RIP1 by A20 in the presence of TNF-α. HuH-7 cells were cotransfected with plasmid pRK5-A20 (or pRK5) and pCMV-HA-Ub (WT), pCMV-HA-Ub (K48) or pCMV-HA-Ub (K63) in the context of TNF-α stimulation. The cell lysates were subjected to immunoprecipitation (IP) with anti-RIP1 antibody followed by Western blot analysis with anti-HA antibody.

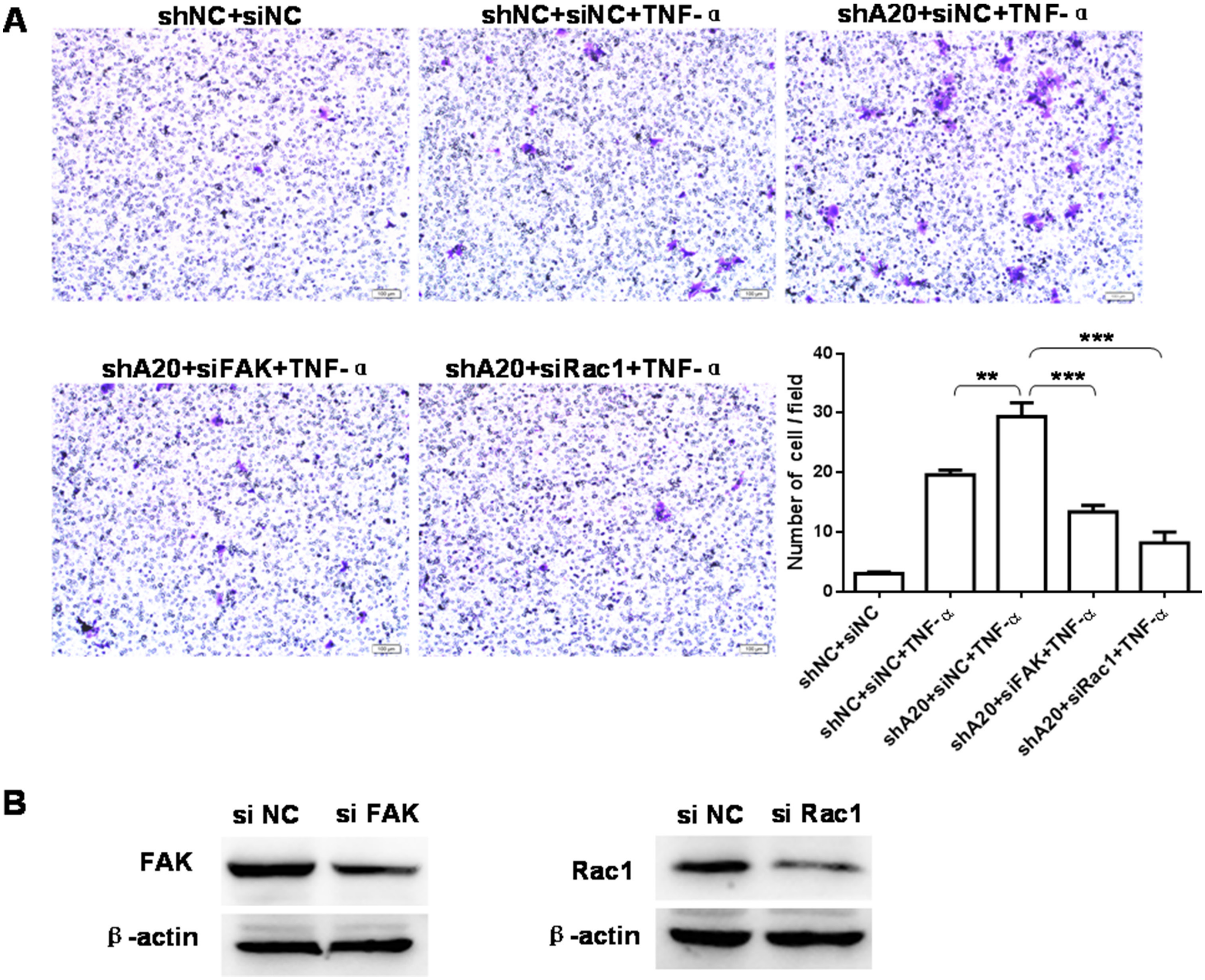

Supplementary Figure S5: Enhancement of HCC cell motility by knockdown of A20 expression was dependent of RAC1 or FAK. (**A**) Hep-3B cells were cotransfected with shNC+siNC, shA20+siNC, shA20+siFAK or shA20+siRAC1 in the presence of TNF-α. The cell motility was evaluated by Transwell migration assay. The number of cells migrated to the bottom surface of the Transwell membrane in the group shA20+siNC was significantly greater than that in the group shNC+siNC . The number of migratory cells in the group shA20+siFAK or shA20+siRAC1 was significantly decreased compared to the group shA20+siNC . ** $p < 0.01$, *** $p < 0.001$. (**B**) Western blot assay showed that the siRAC1 or siFAK silenced the expression of RAC1 or FAK respectively.

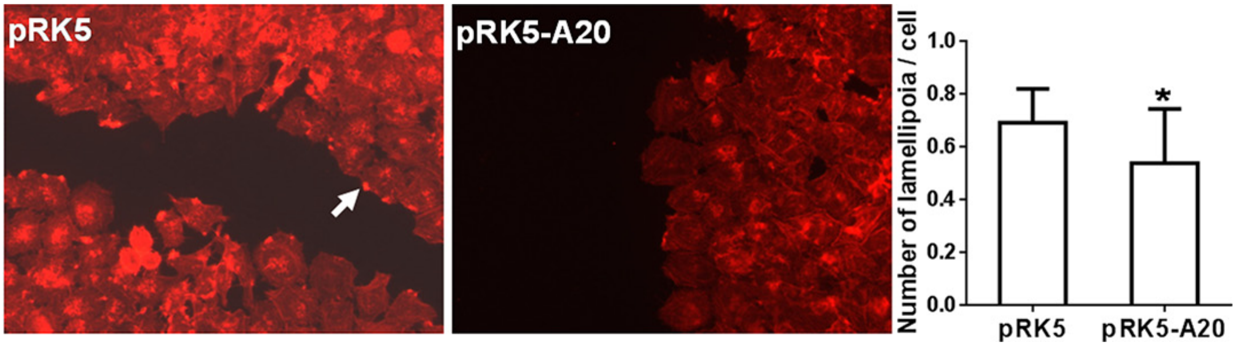

Supplementary Figure S6: A20 overexpression inhibits the formation of ruffling lamellipodia in HCC cells. A scratch was made across the confluent monolayer of BEL7402 cells transfected with pRK5-A20 or pRK5. Fluorescence actin staining was performed. The ruffling lamellipodia in the cells located at the edge of the scratch was counted by fluorescence microscopy. The mean number of the lamellipodia per cell in the pRK5-A20 group was less than that in the pRK5 control group. The white arrow indicates the ruffling lamellipodia. Data are expressed as mean ± SD. *$p < 0.05$.

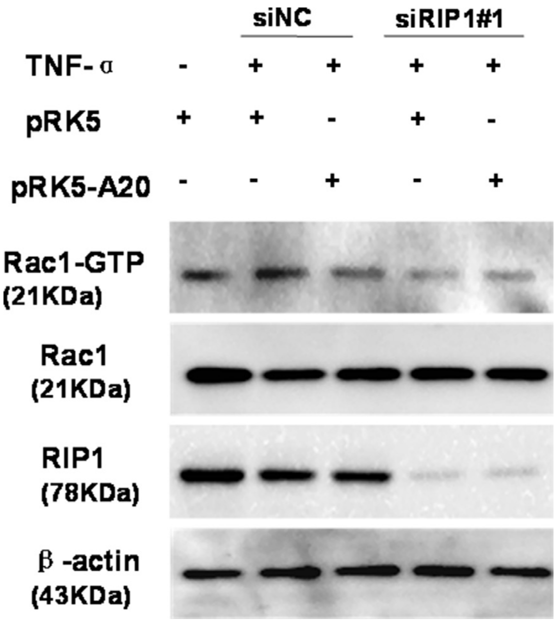

Supplementary Figure S7: The inhibitory effect of A20 overexpression on the RAC1 activation was associated with RIP1. HuH-7 cells were cotransfected with pRK5 or pRK5-A20 plasmid and siNC or siRIP1 followed by TNF-α stimulation. The RAC1 activation was evaluated by RAC1 pull-down assay. Silence of RIP1 by siRIP1 abrogated the inhibition of A20 on the RAC1 activation.
